# Age-Stratified T Cell Responses in Children Infected with *Mycobacterium tuberculosis*

**DOI:** 10.3389/fimmu.2017.01059

**Published:** 2017-09-05

**Authors:** Alexandra Dreesman, Véronique Corbière, Violette Dirix, Kaat Smits, Sara Debulpaep, Iris De Schutter, Myriam Libin, Mahavir Singh, Anne Malfroot, Camille Locht, Françoise Mascart

**Affiliations:** ^1^Laboratory of Vaccinology and Mucosal Immunity, Université Libre de Bruxelles (U.L.B.), Brussels, Belgium; ^2^Department of Pediatrics, CHU Saint-Pierre, Brussels, Belgium; ^3^Department of Pediatric Pulmonology, Cystic Fibrosis Clinic and Pediatric Infectious Diseases, Universitair Ziekenhuis Brussel (UZ Brussel), Brussels, Belgium; ^4^Lionex Diagnostics and Therapeutics, Braunschweig, Germany; ^5^INSERM, U1019, Lille, France; ^6^CNRS, UMR8204, Lille, France; ^7^Université de Lille, Lille, France; ^8^Institut Pasteur de Lille, Centre d’Infection et d’Immunité de Lille, Lille, France; ^9^Immunobiology Clinic, Hôpital Erasme, Université Libre de Bruxelles (U.L.B.), Brussels, Belgium

**Keywords:** tuberculosis, children, interleukin-17, interferon-γ, tumor necrosis factor-α, heparin binding hemagglutinin, early-secreted-antigenic target-6

## Abstract

Tuberculosis (TB) in young children differs from adult TB in that the risk of rapid progression to active TB (aTB) is higher in children than in adults. The reasons for this increased risk are not fully understood. Early differentiation remains difficult between children at risk to develop aTB from those who will remain healthy and develop a latent TB infection (LTBI). Biomarkers to differentiate aTB from LTBI in children, especially in very young children, are urgently needed. To identify *M. tuberculosis-*specific functional T cell subsets related to clinical manifestations in children, we enrolled 87 children exposed to *M. tuberculosis*. After standard clinical assessment, the children were classified as aTB, LTBI, or uninfected. Their CD4^+^ T cell cytokine profiles (IFN-γ, TNF-α, IL-2, IL-17) were analyzed at the single-cell level by flow cytometry after stimulation with three mycobacterial antigens, purified protein derivative (PPD), early-secreted-antigenic target-6 (ESAT-6), or heparin-binding hemagglutinin (HBHA). This approach identified age-related discriminative markers between aTB and LTBI. Whereas among the 3- to 15-year-old children, an excellent discrimination between aTB and LTBI was provided by comparing the ratio between the proportions of ESAT-6-induced IFN-γ^single+^ and ESAT-6-induced TNF-α^single+^CD4^+^ T lymphocytes, this was not the case for children younger than 3 years. By contrast, in this group (<3years), the analysis of HBHA-induced IL-17^single+^CD4^+^ T lymphocytes allowed us to identify children with LTBI by the high proportion of this cellular lymphocyte subset, whereas this was not the case for children with aTB. The analysis at the single-cell level of T cell immune responses induced by mycobacterial antigens are, thus, different in infected children younger or older than 3 years of age. HBHA-induced IL-17 production by CD4^+^ T lymphocytes was associated with protection only in children under 3 years who are at high risk for rapid progression to aTB. This suggests that the HBHA-induced IL-17 production by CD4^+^ T lymphocytes is a potential new correlate of protection against *M. tuberculosis* in humans, and that the distinction between children with LTBI and those with aTB is possible based on age-related diagnostic markers.

## Introduction

Tuberculosis (TB), an infectious disease caused by *Mycobacterium tuberculosis*, remains a global health problem with an estimated 10.4 million incident cases of active TB (aTB) in 2015 (1). Children account for an increasing proportion of TB cases in both high-resource and low-resource countries, with an estimated 1 million incident cases of aTB in children in 2015, leading to 140,000 deaths (1–4). The pathogenesis of pediatric TB remains poorly understood. It differs from that of adult TB by several points and is associated with significant morbidity and mortality (5–7). In contrast to adult TB, pediatric aTB most often results from progression of a primary infection with *M. tuberculosis* rather than from reactivation of a latent infection (5–7). Although children may control the infection and develop a latent TB infection (LTBI), infected infants and young children up to 3 years are at high risk for rapid progression to aTB, which is often severe and disseminated (2). Even though the reasons for this high risk remain incompletely understood, early differential diagnosis between infected children who remain healthy and become LTBI, and those who develop aTB is of utmost importance to adapt the treatment accordingly (i.e. treatment with one or two anti-mycobacterial drugs for LTBI vs. three or more drugs for aTB) (7).

Microbiological diagnosis of aTB in young children remains a challenge, as sputum is difficult to obtain in this age category and the disease is often pauci-bacillary. Therefore, diagnosis often relies on a cluster of epidemiological, clinical, and radiological findings, but the differential diagnosis with LTBI children remains a problem (7–9). To approach this issue, several investigators have compared the immune responses of children with aTB to those of children with LTBI, aiming at identifying biomarkers that could distinguish between these two stages by a blood test. Whereas several studies reported biomarkers able to distinguish uninfected from infected children, only few promising results were obtained on small cohorts of infected children to differentiate aTB from LTBI by the assessment of a T-cell activation marker (10) or by combining measurements of TNF-α and IL-10 concentrations in plasma from purified protein derivative (PPD)-stimulated blood (11), a method that may not be applicable to BCG-vaccinated children.

Similar approaches were taken in adults, where the best differentiation between aTB and LTBI was obtained by the analysis of the IFN-γ secretion induced by the mycobacterial latency antigen heparin-binding hemagglutinin (HBHA) (12, 13), or by flow cytometry analyses of CD4^+^ T lymphocytes producing only TNF-α in response to the early-secreted-antigenic target-6 (ESAT-6) mycobacterial antigen (14). As the first biomarker did not provide a distinction between aTB and LTBI in children (15), we investigated here whether the analysis at the single-cell level of the production of a limited number of cytokines in response to PPD, ESAT-6, or HBHA would provide a useful biomarker to distinguish children with aTB from those with LTBI. In addition to HBHA-specific IFN-γ, a biomarker of LTBI in adults (12, 13), and to ESAT-6-specific TNF-α, a biomarker of aTB (14), we analyzed the IL-2 production, reported as a potential biomarker for protection by several authors (16, 17), and IL-17, whose production was associated with LTBI by some authors and with aTB by others (18–20).

We confirm that the CD4^+^ T cell cytokine response induced by mycobacterial antigens is different in *M. tuberculosis* infected compared to non-infected (NI) children. More importantly, we identified different functional CD4^+^ T lymphocyte subsets associated with clinical protection from aTB in *M. tuberculosis*-infected children according to their age. Whereas HBHA-induced IL-17 production by CD4^+^ T lymphocytes was associated with protection in children under 3 years of age, ESAT-6-induced IFN-γ^single+^CD4^+^ T lymphocytes characterized infected older children who remain healthy. Children with aTB older than 3 years were in contrast characterized by ESAT-6-induced TNF-α^single+^CD4^+^ T lymphocytes.

## Materials and Methods

### Enrolled Children

**Figure 1:**
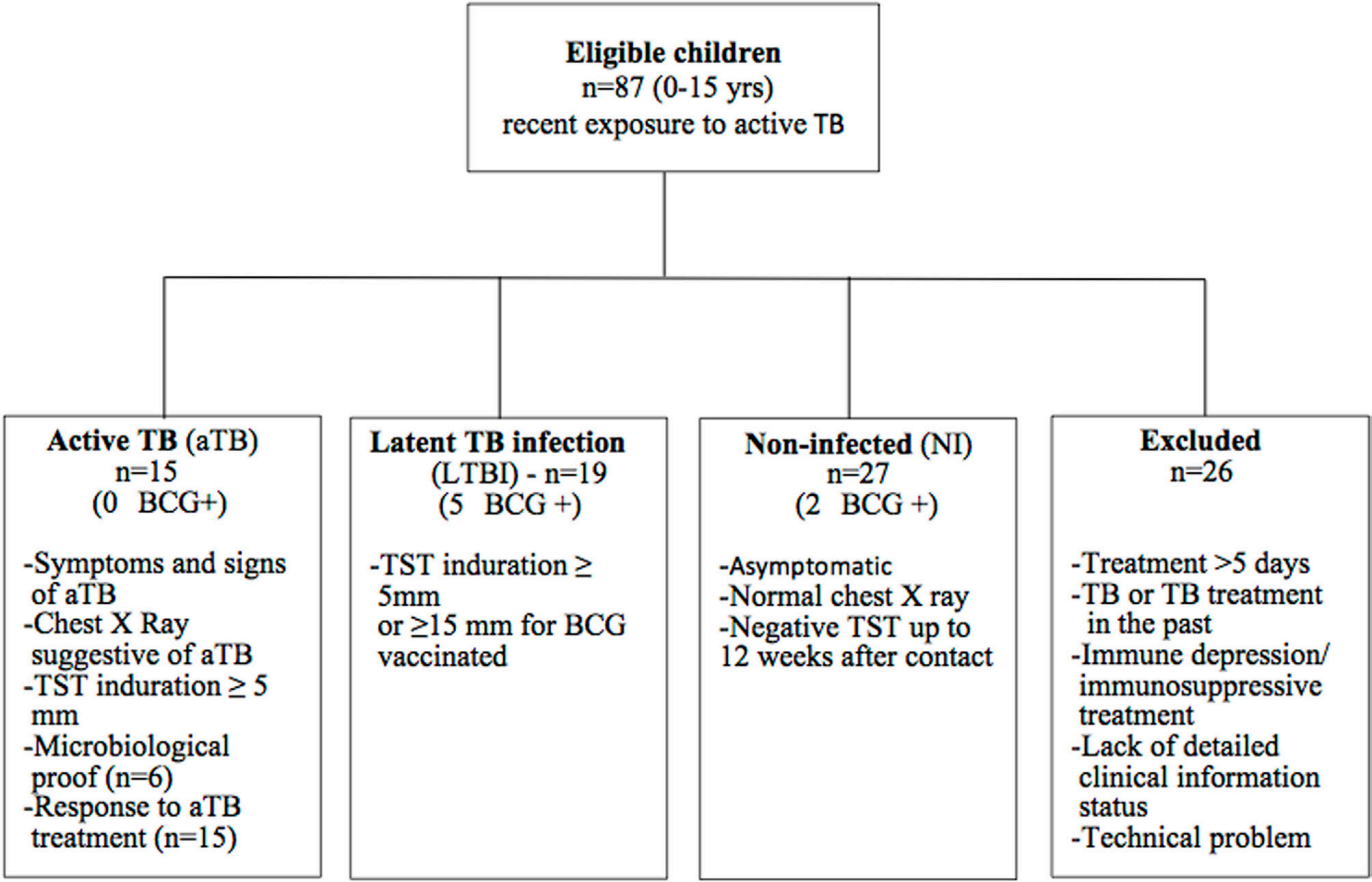
Flowchart of inclusion. Eighty-seven eligible children were prospectively enrolled in the study. Using strict criteria as defined in the Figure, they were retrospectively classified as NI, LTBI, or aTB. Twenty-six children were excluded from the final analysis for reasons explained in the Figure.

Eighty-seven children 0–15 years old, living in a low TB incidence country (Belgium) and recently exposed to an aTB index case (sputum smear positive adult) were prospectively enrolled from three Brussels-based University Hospitals. The pediatricians initially classified the children in three groups: aTB, infected without disease who will become latently infected (LTBI), and probably NI. aTB diagnosis was based on the presence of symptoms and signs consistent with aTB (chronic cough, persistent fever, night sweats, otherwise unexplained weight loss), radiological findings suggestive of aTB, TB exposure history, tuberculin skin test (TST) results (2 IU PPD RT23, tuberculin, PPD; Statens Serum Institute, Copenhagen, Denmark), microbiological results (culture or polymerase chain reaction) and/or response to treatment with anti-tuberculous therapy (Figure 1) (7, 9). LTBI children were defined by a positive TST in an exposed child, without clinical or radiological signs of active disease (7, 9). The positivity of the TST was defined according to the CDC guidelines, as an induration of at least 5 mm, or of at least 15 mm in case of previous BCG vaccination (21). In this study, all included, non-BCG-vaccinated infected children had an induration size of at least 10 mm. Exposed but NI children were all asymptomatic with a TST that remained negative up to 8–12 weeks after last contact with the aTB index case.

### Cell Isolation, Stimulation, and Intracellular Cytokine Staining

Heparinized blood was collected from all eligible children, and the samples were processed within a maximum delay of 4 h after blood drawing. We chose to work on fresh samples based on an earlier study showing a loss of sensitivity using frozen material (22). Peripheral blood mononuclear cells (PBMC) purified by density gradient centrifugation (12) were incubated at 2.10^6^ PBMC/ml during 5 days at 37°C (5% CO_2_), with either (i) 0.5 µg/ml staphylococcal enterotoxin B (SEB) (Sigma-Aldrich, Bornem, Belgium) as a positive control, (ii) 4 µg/ml PPD (Statens Serum Institute, Copenhagen, Denmark), (iii) 10 µg/ml ESAT-6 (Lionex, Diagnostics & Therapeutics GmbH, Braunschweig, Germany), (iv) 10 µg/ml HBHA, purified from *Mycobacterium bovis* BCG as detailed elsewhere (23), or (v) antigen-free medium as a negative control. A long-term stimulation protocol was selected to assure maximum sensitivity, as optimized by Smits et al. (24, 25). Brefeldin A and Monensin (at 3 µg/ml and 2,000-fold final dilution, respectively, both from BD Biosciences, Mountain View, CA, USA) were added during the last 12–14 h of culture. The cells were then stained with viability dye (ThermoFisher Scientific, Waltham, MA, USA, Live/dead Fixable Dead Cells Stain Kit-Aqua, 2.5 µl aqua-H_2_O), fixed and permeabilized (BD Biosciences kit cytofix/cytoperm). They were finally incubated with fluorescence-conjugated monoclonal antibodies directed against different surface antigens (anti-CD3, clone UCHT1, horizon V450 and anti-CD4, clone SK3, APCH7), and intracellular cytokines (anti-IFN-γ, clone B27, APC; anti-TNF-α, clone Mab11, PerCP Cy 5.5; anti-IL-2, clone 5344.111, FITC; anti-IL-17A, clone BL168, PE). Cells were acquired on a FACS Canto II (BD Biosciences) and analyzed with the FACS Diva 6.1.3 Software.

### Data Analysis

Flow cytometry data were analyzed using the FlowJo software (Tree Star, Ashland, OR, USA). Dead cells and cell doublets were excluded (see Figure S1 in the online data supplement for the gating strategy). As no consensus exists in the literature, the threshold of positivity to consider results different from background and to avoid false-positive results was arbitrarily defined using two criteria: the absolute number of cytokine-producing CD4^+^ T cells of at least 100, and the percentage of cytokine-producing cells among CD4^+^ T lymphocytes of at least twice the value obtained for the unstimulated sample. The proportions of antigen-induced cytokine-containing CD4^+^ T lymphocytes were analyzed after subtraction of the results obtained for unstimulated samples, and they were compared between two groups by the Mann–Whitney *U* test (GraphPad Prism Software version 5.04, La Jolla, CA, USA, www.graphpad.com) with *p* values <0.05 being considered as significant. Duplicates were performed for PPD-induced-IFN-γ containing CD4^+^ T lymphocytes on five different blood samples. The correlation between the results obtained for the duplicates for these five samples was excellent (*r*^2^ = 0.99862; not shown). The cytokine profiles based on their co-expression were assessed by Boolean gating, and data sorting and analysis were done with the SPICE software, representing relative frequencies of cells producing all combinations of the four examined cytokines (unequal variances *t*-test) (26).

## Results

### Children Included in the Final Analysis

According to a retrospective analysis of the clinical features and TST results, data from children who did not meet the strictly defined classification criteria (co-existence of a known immune depression or of a concomitant immunosuppressive treatment, intermediate TST results between 5 and 14 mm in a child with a past BCG vaccination, TB treatment in the past, more than 1 week of tuberculostatic treatment, or a lack of sufficiently detailed clinical information), as well as those with an insufficient blood volume for the *in vitro* tests, were excluded from the final analysis (Figure 1). The results from 61 children were thus included in the final analysis. The major demographic and clinical data of these children are provided in Table 1.

**Table 1:** Demographic and clinical data.

	aTB	LTBI	Non-infected
Total numbers	15	19	27
Age (years)			
Mean	4.3	6.7	2.5
Median (range)	2 (0–14)	5 (2–15)	2 (0–8)
Gender			
Male (%)	47	58	60
Female (%)	53	42	40
Ethnicity [number and (%)]			
North-African	7 (47)	6 (32)	12 (44)
Sub-Saharan African	5 (33)	3 (16)	5 (19)
Asiatic	2 (13)	4 (21)	4 (15)
South-American	1 (7)	0	0
European	0	3 (16)	2 (7)
Other/mix/unknown	0	3 (16)	4 (16)
BCG vaccinated [number and (%)]	0	5 (26)	2 (7)
*Mycobacterium tuberculosis* infection status			
TST results			
% Positive	100	100	0
Range of induration (mm) - BCG+	NA	15–20	NA
Range of induration (mm) - BCG−	15–28	12–28	NA
Abnormal chest X-ray (%)	93	0	0
Microbiology			
Smear+ and/or culture+ and/or PCR+ (%)	40	0	0
Response to TB treatment (%)	100	NA	NA
Pulmonary/extra-pulmonary TB (%)	13/2		

*aTB, active tuberculosis; LTBI, latently TB infected; TST, tuberculosis skin test; BCG, bacille Calmette–Guérin; PCR, polymerase chain reaction; NA, non-applicable*.

### Mycobacteria-Specific CD4^+^ T Lymphocyte Cytokine Responses

**Figure 2:**
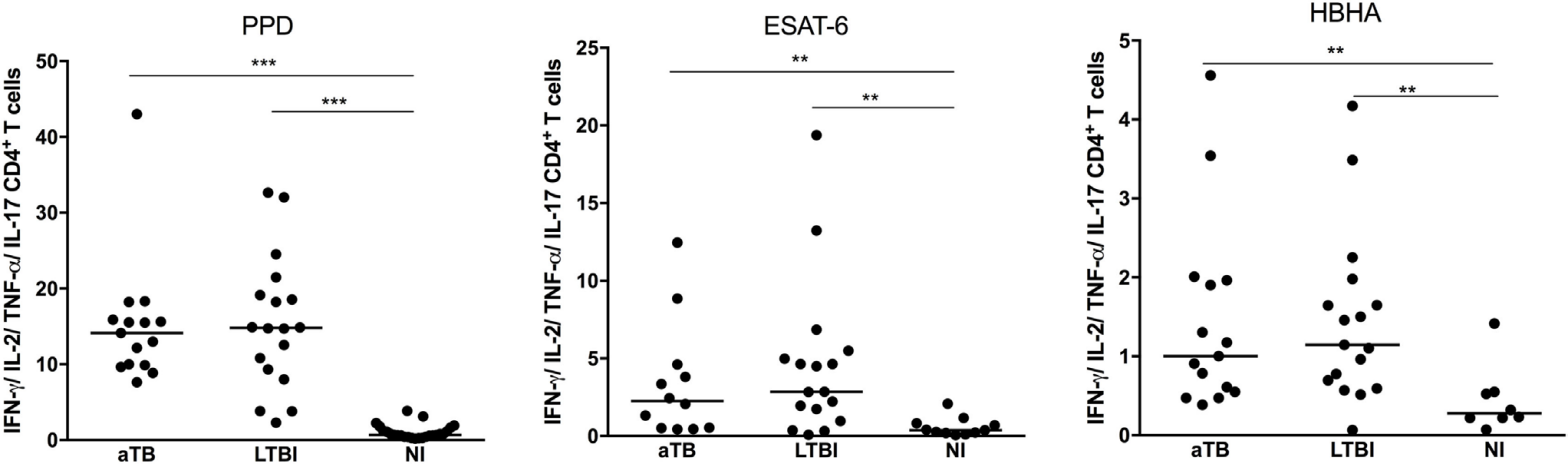
*Mycobacterium tuberculosis*-specific cytokine expression by CD4^+^ T lymphocytes. Peripheral blood mononuclear cells were *in vitro* stimulated during 5 days with purified protein derivative (PPD), early-secreted-antigenic target-6 (ESAT-6), or heparin-binding hemagglutinin (HBHA), and the percentages of CD4^+^ T lymphocytes expressing one or several of the four studied cytokines (IFN-γ, TNF-α, IL-2, IL-17) were measured by flow cytometry. Children with active TB (aTB) were compared to latently TB infected (LTBI) and non-infected (NI) children. Each dot corresponds to the result from an individual child and horizontal bars represent the medians of the results. ***p < 0.001; **p < 0.01.

We first compared the three groups of children for their percentages of antigen-specific CD4^+^ T cell cytokine responses, i.e., all the CD4^+^ T lymphocytes expressing one or several of the four cytokines analyzed, each cytokine being produced alone or in combination with one or several other cytokines. Whereas most infected children had detectable levels of CD4^+^ T lymphocytes producing at least one cytokine in response to the mycobacterial antigens (97% in response to PPD, 85% in response to ESAT-6, and 94% in response to HBHA), NI children had no or very low cytokine responses to these antigens (Figure 2). The percentages of antigen-specific CD4^+^ T lymphocytes containing at least one cytokine were, thus, significantly higher in LTBI and aTB children than in NI children, but they did not discriminate the two groups of infected children (Figure 2). The analysis of the proportions of CD4^+^ T lymphocytes expressing only one cytokine, IFN-γ, TNF-α, IL-2, or IL-17, also did not provide a discrimination between LTBI and aTB children (data not shown).

In contrast to the CD3^+^CD4^+^ lymphocytes, the proportions of cytokine-containing CD3^+^CD4^−^ lymphocytes in response to the three mycobacterial antigens were very low (data not shown) and we, therefore, focused this study on CD4^+^ T lymphocytes.

### Mycobacteria-Specific Cytokine-Producing CD4^+^ T Lymphocyte Subsets

**Figure 3:**
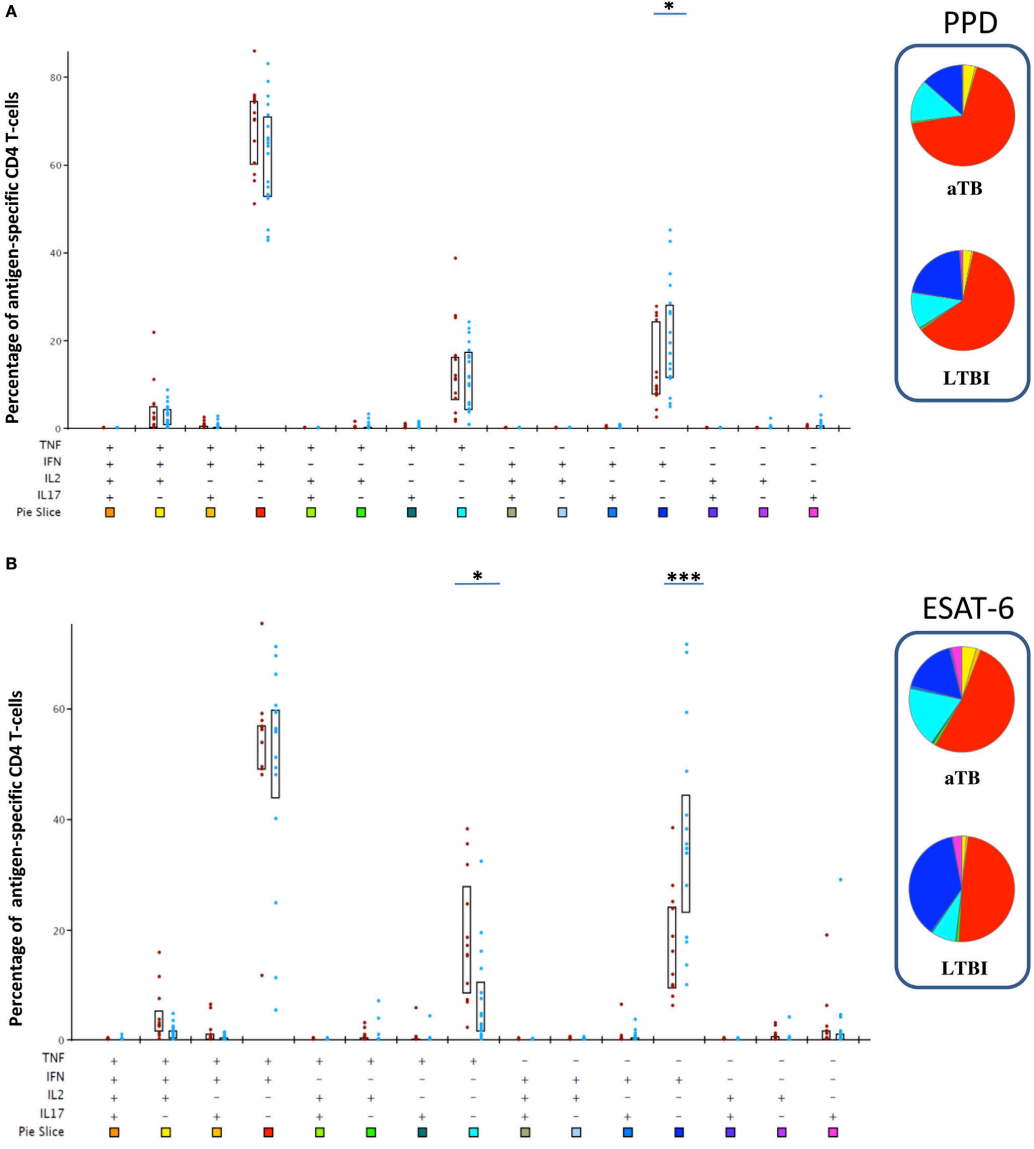

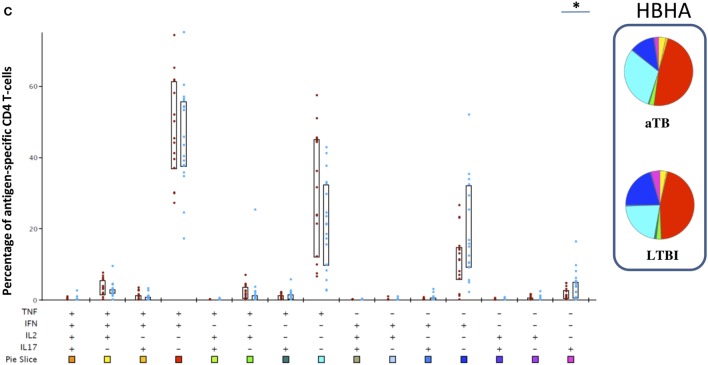
Poly-functional profile of *Mycobacterium tuberculosis*-specific CD4^+^ T lymphocytes after *in vitro* stimulation with purified protein derivative (PPD), early-secreted-antigenic target-6 (ESAT-6), or heparin-binding hemagglutinin (HBHA). Peripheral blood mononuclear cell were *in vitro* stimulated during 5 days with PPD **(A)**, ESAT-6 **(B)**, or HBHA **(C)** and the percentages of all possible combinations of the four studied cytokines (IFN-γ, TNF-α, IL-2, and IL-17) were analyzed among the *M. tuberculosis*-specific cytokine-containing CD4^+^ T lymphocytes. Children with active TB (aTB) are represented by the red dots, whereas latently TB infected (LTBI) children are represented by the blue dots. All the possible combinations of cytokines are represented on the *x*-axis of the bar charts graphs, and color coded, whereas the percentages of each subpopulation within *M. tuberculosis*-specific CD4^+^ T cells are shown on the *y*-axis. Boxes represent interquartile ranges (P25–75). Results were analyzed with SPICE and degrees of significance are based on the “unequal variance *T*-test.” *p < 0.05; ***p = 0.001. The pie charts average the data for both groups (aTB and LTBI), each slice corresponding to the proportion of *M. tuberculosis*-specific CD4^+^ T cells positive for a certain combination of cytokines. The colors in the pie chart are defined by the 15 color boxes on the *x*-axis of the bar chart.

We next focused on the LTBI and aTB children, as strictly defined by the recommended systematic diagnostic approach (7), to assess the quality of the antigen-induced CD4^+^ T cell responses. We characterized within the cytokine-containing CD4^+^ T lymphocytes the 15 different subpopulations co-expressing different combinations of the four analyzed cytokines. Only four CD4^+^ subpopulations were well represented in response to PPD, five in response to ESAT-6, and six in response to HBHA (Figure 3). In response to the three antigens, co-expression of IFN-γ and TNF-α dominated among the possible cytokine combinations, followed by the single expression of IFN-γ or TNF-α, and finally by a subpopulation co-expressing TNF-α, IFN-γ, and IL-2 (Figure 3). HBHA also induced a small proportion of CD4^+^ T lymphocytes co-expressing TNF-α and IL-2, and both ESAT-6 and HBHA induced a small proportion of CD4^+^ T lymphocytes producing only IL-17 (Figure 3).

Notably, most cytokine-producing CD4^+^ T cell subsets did not significantly differentiate LTBI form aTB children, with the following exceptions. Children with aTB had higher proportions of ESAT-6-induced-TNF-α^single+^ cells than LTBI children (*p* < 0.05), and lower proportions of PPD- and ESAT-6- induced IFN-γ^single+^ cells (*p* < 0.05 for PPD and *p* = 0.001 for ESAT-6), as well as lower proportions of HBHA-induced-IL-17^single+^cells (*p* < 0.05) (Figure 3).

### Age-Related Differences in CD4^+^ T Cell Cytokine Profiles

Whereas the above mentioned results indicate a certain level of significant differences in the quality of the CD4^+^ T cell responses between LTBI and aTB children, a high overlap between these two groups of children persisted and the results were rather heterogeneous. As the infected children in this cohort varied by age from 0 to 15 years, and as the risk to progress to active disease is highest in children below 3 years of age (2), this heterogeneity within the immune responses may be due to age differences. We, therefore, analyzed separately the proportions of the most discriminant functional CD4^+^ T lymphocytes subsets for young (up to 3 years old) and older children (more than 3 years old).

**Figure 4:**
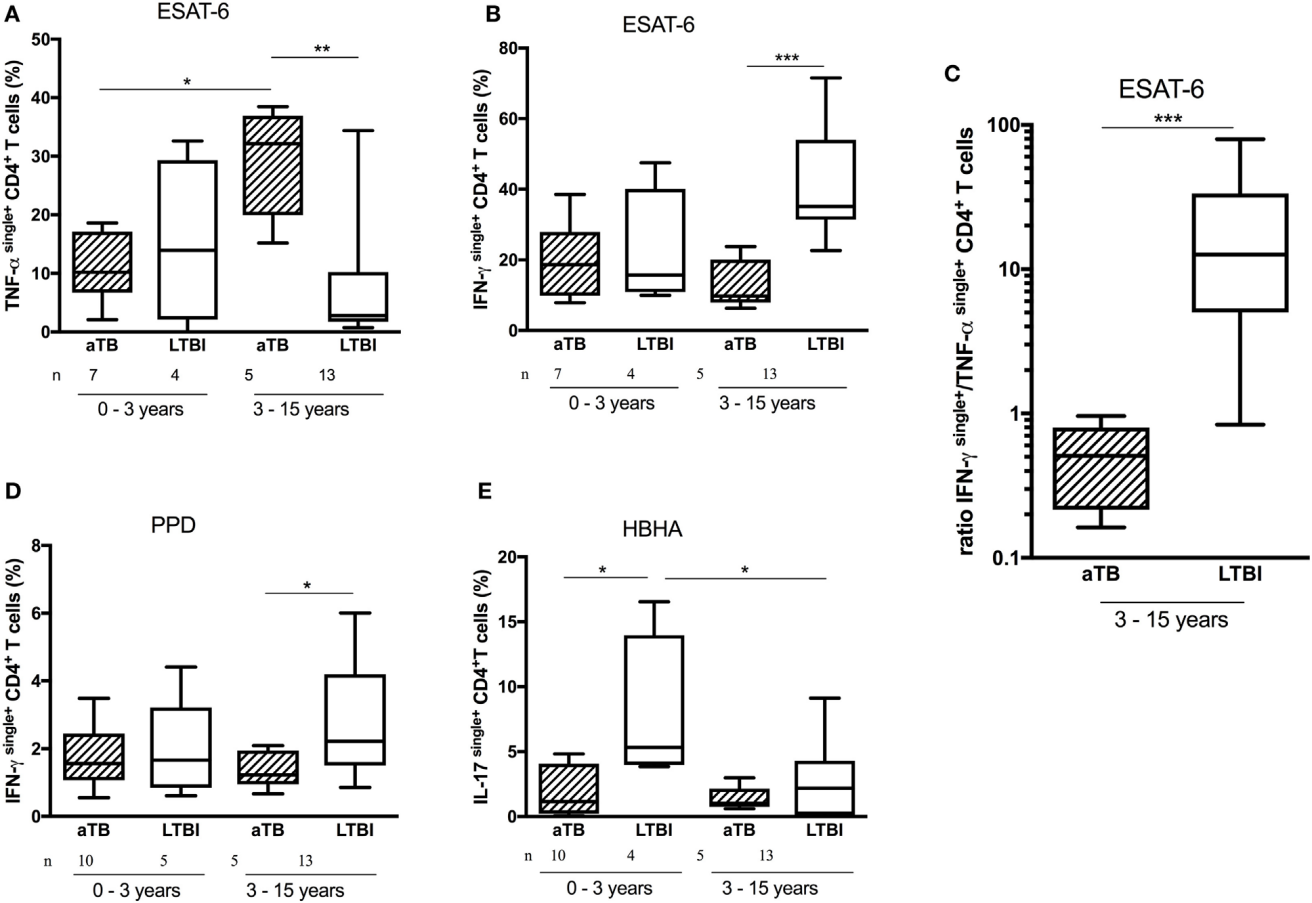
Age-related differences in CD4^+^ T cell cytokine profiles. Peripheral blood mononuclear cells were *in vitro* stimulated during 5 days with early-secreted-antigenic target-6 (ESAT-6), purified protein derivative (PPD), or heparin-binding hemagglutinin (HBHA), and the frequency of cytokine^single+^CD4^+^ T cells was analyzed and compared between infected children who were younger or older than 3 years of age, and between active TB (aTB) (hatched columns) and latently TB infected (LTBI) children (open columns). Among older children, the frequency of ESAT-6-induced TNF-α^single+^CD4^+^ T cells **(A)** was higher and the frequencies of both ESAT-6- and PPD-induced IFN-γ^single+^CD4^+^ T cells were lower **(B,D)** among children with aTB compared to LTBI children. This resulted in lower ratios between the proportions of ESAT-6-induced IFN-γ^single+^ and ESAT-6-induced TNF-α^single+^CD4^+^ T cells in children older than 3 years **(C)**. By contrast, in children younger than 3 years, only the frequency of IL-17^single+^CD4^+^ T cells was higher among LTBI children compared to those with aTB **(E)**. Results are illustrated as medians (horizontal bars), 10th–90th percentiles (boxes) and ranges. ***p < 0.005; **p ≤ 0.01; *p < 0.05.

It appears from this analysis that differences found between the two groups of infected children for PPD- and ESAT-6-induced responses were restricted to the 3- to 15-year-old children. In this age category, children with aTB had higher proportions of ESAT-6-induced TNF-α^single+^ cells (*p* = 0.01) and lower proportions of ESAT-6-induced IFN-γ^single+^CD4^+^ T lymphocytes (*p* < 0.005) than LTBI children (Figures 4A,B). The ratio between the proportions of ESAT-6-induced IFN-γ^single+^ and ESAT-6-induced TNF-α^single+^CD4^+^ T lymphocytes provided an excellent discrimination between LTBI and aTB children older than 3 years (*p* < 0.005) (Figure 4C). Such differences were not observed for younger children as, compared to the older children, they had significantly lower proportions of ESAT-6-induced TNF-α^single+^ cells when suffering from aTB (*p* < 0.05) (Figure 4A). Children older than 3 years with aTB were also characterized by lower proportions of PPD-induced IFN-γ^single+^CD4^+^ T lymphocytes compared to LTBI children (*p* < 0.05) (Figure 4D).

In contrast to the older children, among children below 3 years of age the only discriminant functional CD4^+^ T lymphocyte subset between LTBI and aTB children was the HBHA-induced IL-17^single+^CD4^+^ T lymphocyte subset (Figure 4E). The proportion of these cells was significantly higher among LTBI than among aTB children (*p* = 0.036), allowing a rather good discrimination between these two groups of young infected children (Figure 4E).

## Discussion

Infants and young children are highly susceptible to severe and disseminated *M. tuberculosis* infection, which may quickly lead to death. Therefore, early identification and treatment of aTB in children is of utmost importance. Whereas the identification of *M. tuberculosis*-infected children, at least in non-vaccinated children, is feasible by the detection of the immune responses to mycobacterial antigens (TST and/or mycobacteria-specific cytokine responses) (27, 28), the differential diagnosis between aTB and LTBI children remains difficult. Yet, this distinction is clinically very important, as it determines the treatment regimen. First promising results were recently reported for a small cohort of children older than 3 years by combining the measurement of different cytokines released by stimulation of blood cells with PPD (11). We report here for the first time that the immune response of infected children who do not develop aTB and, hence, appear to be protected by their immune response, is different between children younger than 3 years and older children. Young LTBI children mount an IL-17^+^CD4^+^ T lymphocyte response to the mycobacterial latency antigen HBHA, whereas young children with aTB and older children do not. The limitation of this study is the low number of LTBI children in this age category although 19 LTBI children were included in the final analysis of the results. This is a direct consequence of the high susceptibility of young children to develop aTB when infected with *M. tuberculosis* and indicates that the population analyzed here is well representative of the global epidemiology of *M. tuberculosis* infection in low TB incidence countries. By contrast, only a minority of children with aTB were included in the 3- to 15-year-old group. In spite of the relatively low numbers of children who could be included in the final analyses, the differences were very striking and highly significant.

HBHA is a protective antigen against *M. tuberculosis* in a mouse model of infection (29, 30). Optimal protection was associated with the combined induction by HBHA of IFN-γ and IL-17, indicating the role of HBHA-induced IL-17 in protection, in addition to the already known role of HBHA-induced IFN-γ (30). In humans, the potential role of IL-17 in protection against TB disease remains controversial. Induction of IL-17 by mycobacterial antigens was associated in some studies with protection of LTBI subjects from overt TB disease (19), and uncontrolled *M. tuberculosis* infections were associated with a defect of *M. tuberculosis*-specific CD4^+^ T cells to produce IL-17 (18, 31). By contrast, other studies reported increased IL-17 production in patients with aTB (20, 32). We show here that IL-17 induction by the protective antigen HBHA is associated with protection in young children known to be limited in their ability to produce IFN-γ as a consequence of the decreased capacity of their dendritic cells to produce IL-12p70 (33). However, young children have the capacity to produce IL-23 (34), a cytokine able to induce IL-17 synthesis by polyclonally activated naïve human T cells (35) or by naïve murine T cells activated by *M. tuberculosis*-stimulated dendritic cells (36). Accordingly, our results, therefore, suggest that the IL-17 response to HBHA during *M. tuberculosis* infection could be initiated by IL-23 in the absence of IL-12p70 in young children, similar to what was reported in mice (36). The demonstration of the capacity of HBHA to induce IL-17 synthesis by CD4^+^ T lymphocytes in the very young *M. tuberculosis*-infected children, therefore, points toward a new protective mechanism against TB disease when IFN-γ production is suboptimal. The IL-23/IL-17 pathway may, thus, compensate for the deficiency in IFN-γ production in these very young children and potentially *vice versa* in older children and adults. This reciprocal compensatory mechanism may also explain why numerous cases of susceptibility to mycobacteria are reported in humans lacking IL-12p40 or IL-12Rβ1, and not in those deficient in IL-12p35, the subunit not shared between IL-12 and IL-23 (37). Our results also open the way for the evaluation of new diagnostic tests for early differentiation between aTB and LTBI in young children.

We, thus, show here that *M. tuberculosis*-induced immune responses in children are age-stratified. After 3 years of age, LTBI and aTB children can be easily differentiated by the ratio of ESAT-6-induced IFN-γ^single+^ to TNF-α^single+^CD4^+^ T lymphocytes. This results from the high proportion of ESAT-6-induced TNF-α^single+^ and the low proportion of ESAT-6-induced IFN-γ^single+^CD4^+^ T lymphocytes among >3-year-old children with aTB. The induction by ESAT-6 of TNF-α^single+^CD4^+^ T lymphocytes was reported to be a surrogate marker of aTB in adults (14), and we show here that this functional CD4^+^ T lymphocyte subset is also associated with aTB in children older than 3 years, whereas ESAT-6-induced IFN-γ^single+^CD4^+^ T lymphocytes are associated with LTBI in this age category. This is in contrast to previous reports showing high ESAT-6-induced IFN-γ concentrations released after an *in vitro* stimulation of peripheral blood mononuclear cells or whole blood from both LTBI and aTB children, with even higher IFN-γ concentrations measured in this latter group (11, 38). However, the source of this IFN-γ production was not determined in these studies, whereas our study shows the importance specifically of the IFN-γ^single+^CD4^+^ T lymphocytes. In our study, most IFN-γ induced by ESAT-6 was produced by double-positive IFN-γ^+^TNF-α^+^CD4^+^ T lymphocytes, but this cell subset did not allow us to differentiate LTBI from aTB children (Figure 3B). These findings, thus, highlight the interest to analyze the cytokine production at the functional single-cell level by flow cytometry, as done here, instead of measuring total IFN-γ concentrations released by all cell subsets secreting IFN-γ in response to ESAT-6.

We conclude that the analysis of cytokine profiles in *M. tuberculosis*-specific CD4^+^ T lymphocytes by polychromatic flow cytometry can be considered as a major immunological measure able to discriminate between LTBI and aTB children, but that functional lymphocyte subsets of interest are age-related. We also identified a potential new correlate of protection against *M. tuberculosis* infection in humans, especially in very young children: the HBHA-induced IL-17 production by CD4^+^ T lymphocytes. In practical terms, the determination of functional T cell subsets by flow cytometry can advantageously be performed for single individual, providing rapid read-outs, in contrast to multiple cytokine concentrations measurements that usually are performed by batches for logistic and economical reasons, thereby often delaying the read-outs.

## Ethics Statement

The study protocols (numbers P2011/113 and A2012/051) were approved by the ethics committee ULB-Hôpital Erasme, Brussels, Belgium, and informed written consent was obtained from all parents.

## Author Contributions

AD acquired clinical data, conducted the analyses, interpreted the data, drafted the initial manuscript, and approved the final manuscript as submitted; VC, VD, and KS designed the study, contributed to the optimization of the study protocol, interpreted the data, critically reviewed the manuscript, and approved the final manuscript as submitted; SD, IDS, and AM recruited patients and approved the final manuscript as submitted; ML acquired the data and approved the final manuscript as submitted; MS provided antigen, revised the manuscript, and approved the final manuscript as submitted; CL contributed to the conceptualization of the study and the data interpretation, provided antigen, critically reviewed the manuscript, and approved the final manuscript as submitted; FM conceptualized the overall study, interpreted the data, reviewed and revised the manuscript, and approved the final manuscript as submitted.

## Conflict of Interest Statement

The authors declare that the research was conducted in the absence of any commercial or financial relationships that could be construed as a potential conflict of interest.

## Supplementary Material

Figure S1 Gating strategy applied to evaluate the functional subsets of CD4^+^ and CD4^–^ T lymphocytes based on the intracellular detection of 4 cytokines. Sequential gating was done on time of acquisition (a), live cells (b), single cells (c), lymphocytes (d), CD3^+^ lymphocytes (e), CD3^+^ CD4^+^ (or CD4^–^)T cells (f), cytokines (g–i). The respective percentages of all 15 functional subsets resulting from all possible combinations of the 4 studied cytokines were calculated by boolean gating.
