# Supplementary figures and images for: Age-Stratified T Cell Responses in Children Infected with Mycobacterium tuberculosis
Source: Front Immunol. 2017 Sep 5;8:1059. doi: 10.3389/fimmu.2017.01059 (PMC5591888; doi:10.3389/fimmu.2017.01059)

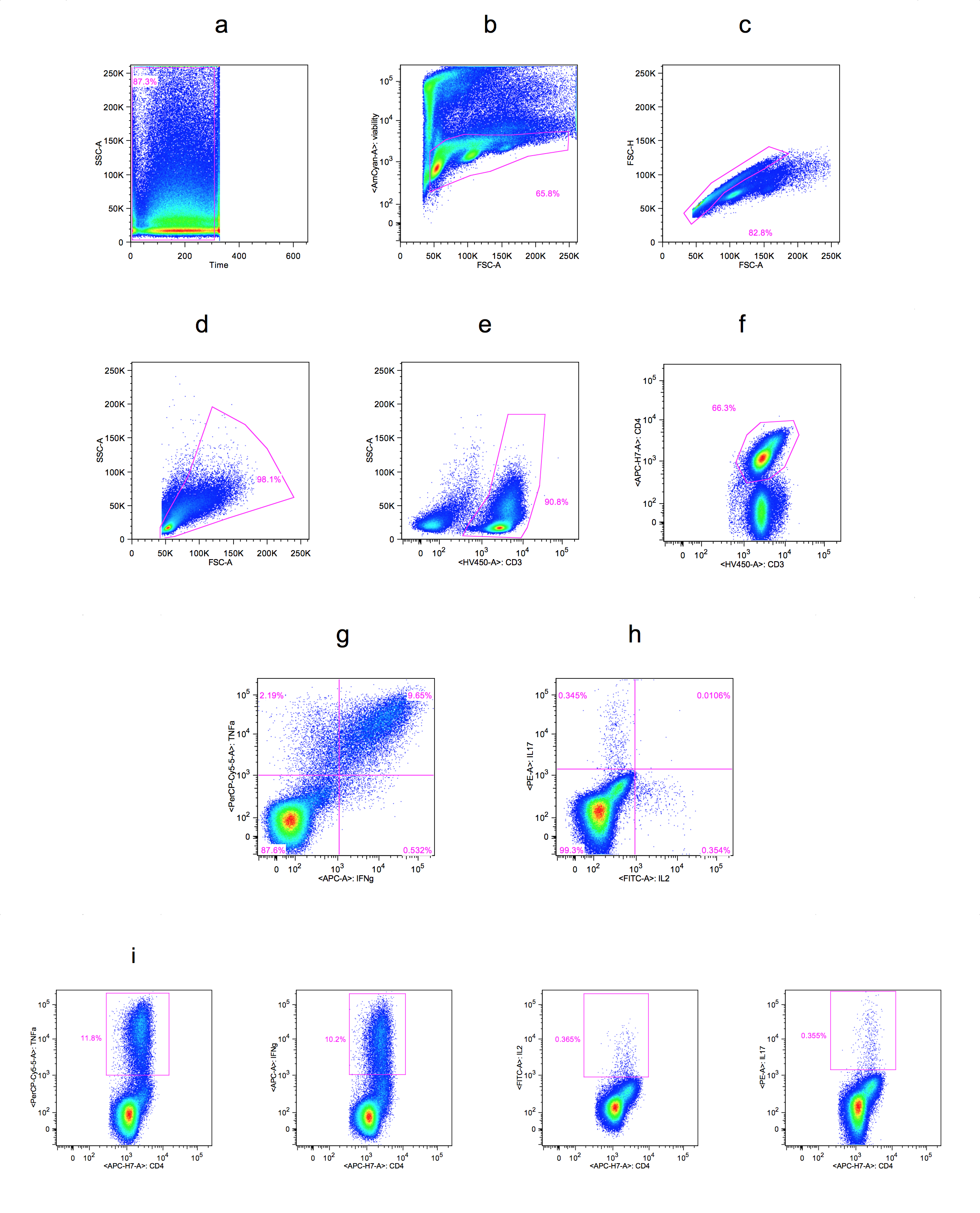

Supplement: Figure S1 — Gating strategy applied to evaluate the functional subsets of CD4+ and CD4– T lymphocytes based on the intracellular detection of 4 cytokines. Sequential gating was done on time of acquisition (a), live cells (b), single cells (c), lymphocytes (d), CD3+ lymphocytes (e), CD3+ CD4+ (or CD4–)T cells (f), cytokines (g–i). The respective percentages of all 15 functional subsets resulting from all possible combinations of the 4 studied cytokines were calculated by boolean gating. [file Image_1.TIFF]
